# Supplementary material for: RNA-Seq analysis of genes affected by Cyclophilin A/DIAGEOTROPICA (DGT) in tomato root development
Source: F1000Res. 2020 Sep 28;9:1175. [Version 1] doi: 10.12688/f1000research.25750.1 (PMC7570327; doi:10.12688/f1000research.25750.1)
Supplement: Peer review history

# Peer review: Reviewer response for version 1

**DOI:** 10.5256/f1000research.28419.r72162

**Published:** 2020-10-16

**Authors:** Aurélien Bailly

**Affiliations:** 1 Department of Plant and Microbial Biology, University of Zurich, Zurich, Switzerland

Ivanchenko and colleagues describe here a transcriptomics data set profiling gene expression in root tips of the tomato mutant *dgt* and matching wildtype. As for several plant immunophilins, loss of function of DIAGETROPICA triggers dramatic structural and behavioural phenotypes in tomato. The link between immunophilins and hormonal control has been extensively recognised; however, the detailed molecular mechanisms behind them remain elusive.

 The interest in this RNAseq dataset lies within the dissection of the root into the three standard spatio-temporal zones of development. Gaining insight into the effect of the *dgt* mutation in the transcriptomes of the meristematic, elongation and mature zones of the root would potentially lead to the isolation of long-sought components of root growth and development machineries.

 The work presented here is technically sound and used state of the art transcripts-reading methods. Data deposition was correctly performed and gene lists produced after alignments and differential expression analysis through regular scripts are clear and readily exploitable. Although the level of variation between replicates, as mentioned by authors, do vary in the different root zones samples, I trust that they separate enough in PCA to yield interesting outcomes. In addition, the relatively reduced number of differentially expressed genes filtered in this analysis will allow for a rapid identification of proteins associated with the dgt phenotype, perhaps outside of the classical pool of auxin-related genes.

 I would express only two regrets: 1) the DGT protein was shown to hold a conserved auxin signalling function down to moss, but it is not mentioned here; and 2) other immunophilins present overlapping roles with DGT, a paradigm worth mentioning somewhere in the introduction.

Are sufficient details of methods and materials provided to allow replication by others?

Yes

Is the rationale for creating the dataset(s) clearly described?

Yes

Are the datasets clearly presented in a useable and accessible format?

Yes

Are the protocols appropriate and is the work technically sound?

Yes

Reviewer Expertise:

Plant Biology

I confirm that I have read this submission and believe that I have an appropriate level of expertise to confirm that it is of an acceptable scientific standard.

# Peer review: Reviewer response for version 1

**DOI:** 10.5256/f1000research.28419.r72160

**Published:** 2020-10-12

**Authors:** Joseph Dubrovsky, Gustavo Rodríguez-Alonso

**Affiliations:** 1 Department of Plant Molecular Biology, Institute of Biotechnology, Universidad Nacional Autónoma de México, Cuernavaca, Mexico; 2 Department of Plant Molecular Biology, Institute of Biotechnology, Universidad Nacional Autónoma de México, Cuernavaca, Morelos, 62210, Mexico

The article by Maria Ivanchenko *et al*. “RNA-Seq analysis of genes affected by Cyclophilin A/DIAGEOTROPICA (DGT) in tomato root development” presents the results of RNAseq analysis of the roots of a tomato mutant affected in Cyclophilin A/DIAGEOTROPICA (DGT) vs wild type. The authors compare three developmental zones in the root tip of mutant and wild type seedlings and identify differentially expressed genes. Considering the pleiotropic effect of the mutation and its relation to auxin signaling and transport, this work is potentially important to understand abnormalities in the root development in the *dgt *mutant and the role of *DGT* in root development. Considering that the formation of lateral root primordia is impaired in *dgt* mutants, this analysis can also be useful in the identification of candidate genes involved in lateral root initiation.

 All the raw data are publicly available, the same as the scripts used for the RNA-seq analysis. A minor suggestion: the authors should explicitly state whether any pre-processing steps were performed on the raw sequencing reads, such as removal of low-quality bases, adapter filtering, or trimming. Regarding the PCA analysis, one of the WT replicates does not group closely with the other WT samples. It could be useful if the authors provide some insight on why this occurs and discuss the effect of this on TPM variation among replicates.

Are sufficient details of methods and materials provided to allow replication by others?

Partly

Is the rationale for creating the dataset(s) clearly described?

Yes

Are the datasets clearly presented in a useable and accessible format?

Yes

Are the protocols appropriate and is the work technically sound?

Yes

Reviewer Expertise:

JD: Plant development, root development, root growth and branching, lateral root development. GR-A: Transcriptomics and evolutionary biology.

We confirm that we have read this submission and believe that we have an appropriate level of expertise to confirm that it is of an acceptable scientific standard.
